# Prognostic Factors in Patients With Locally Advanced Hypopharyngeal Squamous Cell Carcinoma After Radical Radiotherapy/Chemoradiotherapy

**DOI:** 10.1002/cam4.71390

**Published:** 2025-11-20

**Authors:** Xinyue Qu, Zhen Zhao, Yunfan Wu, Rong Qiu, Meng Song, Zhuofan Wang, Qi Liu, Yan Zhao, Yuxiang Wang

**Affiliations:** ^1^ Department of Radiation Oncology, The Fourth Hospital of Hebei Medical University & Hebei Clinical Research Center for Radiation Oncology Shijiazhuang China; ^2^ Department of Otolaryngology, Head and Neck Surgery The Fourth Hospital of Hebei Medical University & Hebei Clinical Research Center for Radiation Oncology Shijiazhuang China

**Keywords:** chemotherapy, locally advanced hypopharyngeal carcinoma, radiotherapy, survival

## Abstract

**Objective:**

To analyze treatment efficacy and prognostic factors in patients with locally advanced hypopharyngeal squamous cell carcinoma (LA‐HPSCC) treated with radical radiotherapy (RT)/chemoradiotherapy (CRT).

**Methods:**

We retrospectively enrolled LA‐HPSCC patients treated with radical RT or CRT during 2015–2021. Clinical and inflammatory factors affecting treatment efficacy were analyzed using SPSS (*v*27).

**Results:**

In all, 193 patients (179 male and 14 female patients) were enrolled. The 1‐year, 3‐year, and median overall survival (OS) were 78.2%, 41.1%, and 28.9 months, respectively. The 1‐year, 3‐year, and median progression‐free survival (PFS) were 69.9%, 34.7%, and 21.8 months, respectively. Univariate analysis showed that second primary cancer, albumin‐fibrinogen ratio (AFR), platelet‐lymphocyte ratio (PLR), neutrophil‐lymphocyte ratio (NLR), clinical T (cT) stage, TNM stage, treatment modality, and short‐term treatment response were associated with OS (*p* < 0.05), while AFR, PLR, cT stage, treatment modality, and short‐term response were associated with PFS (*p* < 0.05). Multivariate analysis revealed AFR and short‐term response as independent risk factors for OS and PFS, and cT and second primary cancer as independent risk factors for OS. Compared with those not receiving concurrent chemoradiotherapy (CCRT), those treated with CCRT had improved OS and PFS if they were aged ≥ 70 years, or had N2/N3 or stage III/IVA disease (*p* < 0.05).

**Conclusions:**

OS and PFS were higher in patients with early cTNM, high AFR, low PLR, CCRT, and good short‐term response. Patients with low NLR had higher OS and a higher incidence of second primary cancers (commonly esophageal and lung cancers).

**Abbreviations**
AC: adjuvant chemotherapy
AFR: albumin‐fibrinogen ratio
AJCC: American Joint Committee on Cancer
CCRT: concurrent chemoradiotherapy
cN: clinical N
CR: complete remission
CRT: chemoradiotherapy
CSCO: Chinese Society of Clinical Oncology
cT: clinical T
CTV1: clinical target volume 1
EP: etoposide‐cisplatin
FLP: fluorouracil‐leucovorin‐platinum
GP: gemcitabine‐platinum
GTV: gross tumor volume
HPC: hypopharyngeal cancer
HPSCC: hypopharyngeal squamous cell carcinoma
IC: induction chemotherapy
IMRT: intensity‐modulated radiotherapy
NCCN: National Comprehensive Cancer Network
NCDB: National Cancer Database
NLR: neutrophil‐lymphocyte ratio
PD: progressive disease
PF: platinum‐fluorouracil
PGTV: planning gross tumor volume
PR: partial remission
PTV1: planning target volume 1
RECIST: Response Evaluation Criteria in Solid Tumors
ROC: receiver operating characteristic
SCCHN: squamous cell carcinoma of the head and neck
SD: stable disease
SEER: Surveillance, Epidemiology, and End Results
SPC: second primary carcinoma
TC: taxane‐carboplatin
TP: taxane‐platinum
TPF: taxane‐platinum‐fluorouracil

## 1 Introduction

Hypopharyngeal cancer (HPC) is a rare type of cancer accounting for approximately 3%–5% of head‐and‐neck malignancies [1]. The main pathological type of HPC is squamous cell carcinoma, and the sites most prone to developing HPC are the pyriform sinus, posterior pharyngeal wall, and posterior annular area. Owing to the deep anatomical location of the hypopharynx, HPC is often not easy to detect in the early stages, and most patients already have locally advanced cancer when their symptoms become apparent [2]. Newman et al. [3] searched the Surveillance, Epidemiology, and End Results (SEER) database, and identified 6647 patients with hypopharyngeal squamous cell carcinoma (HPSCC) between 1973 and 2003. The authors found that the overall survival (OS) of HPSCC patients improved during this time period. The average 5‐year OS improved from 37.5% pre‐1990 (1973–1989) to 41.3% post‐1990 (1990–2003). This improvement was mainly attributed to a significant increase in the use of radiotherapy (RT) regimens without surgery (from 43.1% to 52.1%). The use of RT alone increased 5‐year OS (from 27.6% pre‐1990 to 37.8% post‐1990). In contrast, the use of surgery alone has declined from 14% to 7.3%, and patients treated with surgical therapy alone experienced decreased 5‐year OS (from 49.3% pre‐1990 to 44.5% post‐1990) [3]. Another retrospective study of data from the National Cancer Database (NCDB) analyzed the use of intensity‐modulated radiotherapy (IMRT) and non‐IMRT techniques in 3928 patients with HPSCC. The results showed that the use of IMRT significantly improved survival (5‐year OS: 41.9% in the IMRT group vs. 36.8% in the non‐IMRT group) [4].

According to the guidelines of the National Comprehensive Cancer Network (NCCN) and the Chinese Society of Clinical Oncology (CSCO), patients with locally advanced HPC who are not candidates for surgery should be recommended chemoradiotherapy (CRT) or CRT combined with targeted therapy [5, 6]. Several studies have shown that inflammatory factors are related to the efficacy of RT for head‐and‐neck tumors [7, 8]. Therefore, the present retrospective study aimed to comparatively analyze the treatment efficacy and prognostic factors in patients with locally advanced HPSCC (LA‐HPSCC) treated with radical RT vs. CRT.

## 2 Materials and Methods

### 2.1 Subject Selection

We conducted a retrospective review of patients with LA‐HPSCC who underwent radical RT or CRT in the Fourth Hospital of Hebei Medical University between January 2015 and December 2021. The inclusion criteria were as follows: (1) diagnosis of LA‐HPSCC, defined as stage III–IVB according to the 8th edition of the American Joint Committee on Cancer (AJCC) standard [3]; (2) pathological tumor subtype identified as squamous cell carcinoma; (3) absence of surgical treatment; (4) treatment with RT or CRT; and (5) complete data. Patients meeting any of the following criteria were excluded: stage I–II or stage IVC HPC, surgical resection of the primary tumor or metastatic lymph nodes, non‐squamous cell carcinoma, and incomplete data. The study was approved by the ethics committee of the Fourth Hospital of Hebei Medical University (2022KY262), Shijiazhuang, China.

### 2.2 Treatments

#### 2.2.1 Radiotherapy

IMRT was used for all patients. Gross tumor volume (GTV) was defined as the hypopharyngeal tumor, and GTV‐n was defined as the metastatic lymph nodes in the neck. Clinical target volume 1 (CTV1) included GTV, GTV‐n, and adjacent high‐risk lymphatic drainage areas, while CTV2 included low‐risk lymphatic drainage areas. Planning target volume 1 (PTV1) and PTV2 were obtained by extending CTV1 and CTV2 by 3–5 mm, respectively. Planning gross tumor volume (PGTV) and PGTV‐n were obtained by extending GTV and GTV‐n by 5 mm, respectively. The organs at risk included the right and left parotid glands (V30 < 50%), lens (< 8 Gy), optic nerve (< 54 Gy), brainstem (< 54 Gy), and spinal cord (< 45 Gy). The dose limits for the organs at risk were individualized according to the patients' conditions.

#### 2.2.2 Chemotherapy

Chemotherapy was administered as induction chemotherapy (IC), adjuvant chemotherapy (AC) after IMRT, and concurrent chemoradiotherapy (CCRT). The primary regimens were platinum‐based dual‐drug chemotherapy regimens. The platinum‐based agents used included cisplatin, carboplatin, oxaliplatin, nedaplatin, and lobaplatin, and these were typically combined with drugs such as docetaxel, paclitaxel, nab‐paclitaxel, fluorouracil, tegafur, gemcitabine, or vincristine.

### 2.3 Follow‐Up

The last date of the follow‐up period was October 31, 2022. The follow‐up methods included collection of inpatient medical records, outpatient system inquiries, and telephone follow‐ups. The follow‐up contents encompassed the patients' general condition (dietary habits, changes in physical strength and body weight, regular re‐examinations, etc.), time and location of recurrence or distant metastasis, post‐recurrence/metastasis treatment, survival status, and time of death. Hematological parameters were obtained by collecting peripheral venous blood samples from the patients within 2 weeks before treatment, and used to calculate the neutrophil‐lymphocyte ratio (NLR), platelet‐lymphocyte ratio (PLR), and albumin‐fibrinogen ratio (AFR). Short‐term efficacy was evaluated within 1 month after the completion of all treatments, by using the Response Evaluation Criteria in Solid Tumors (RECIST) version 1.1. The results were classified as follows: (1) complete remission (CR), complete disappearance of all target lesions, with the short axis of all pathological lymph nodes reduced to < 10 mm; (2) partial remission (PR), ≥ 30% decrease in the sum of the longest diameters of all target lesions as compared to the baseline; (3) stable disease (SD), target lesion status between PR and disease progression; and (4) progressive disease (PD), ≥ 20% relative increase in the sum of the diameters of all lesions as compared to the smallest recorded value, with an absolute increase of ≥ 5 mm (emergence of new lesions also indicates PD). OS was defined as the duration from the initial treatment to the date of the patient's death or last follow‐up. Progression‐free survival (PFS) was defined as the duration from the initial treatment to disease progression (including local/regional recurrence or distant metastasis) or death. A second primary cancer at initial diagnosis included other malignancies existing prior to the HPC diagnosis. Second primary cancers during and after the initial diagnosis included other malignancies occurring at any time from before the diagnosis of HPC to the last date of follow‐up. The follow‐up duration was measured in months. Cases lost to follow‐up were censored, with survival time calculated based on the last follow‐up date.

### 2.4 Statistical Analysis

Statistical analyses were carried out using SPSS v27.0 software. Categorical data were evaluated using the chi‐square test. Survival analysis was performed using the Kaplan–Meier method, and differences in survival data were assessed using the log‐rank test. Multivariate analysis was conducted using the Cox proportional hazards regression model. Receiver operating characteristic (ROC) curves were constructed to identify the optimal cutoff values of key nutritional and immune indicators as prognostic markers. Differences were considered statistically significant at *p* < 0.05.

## 3 Results

### 3.1 Clinical Characteristics

**FIGURE 1:**
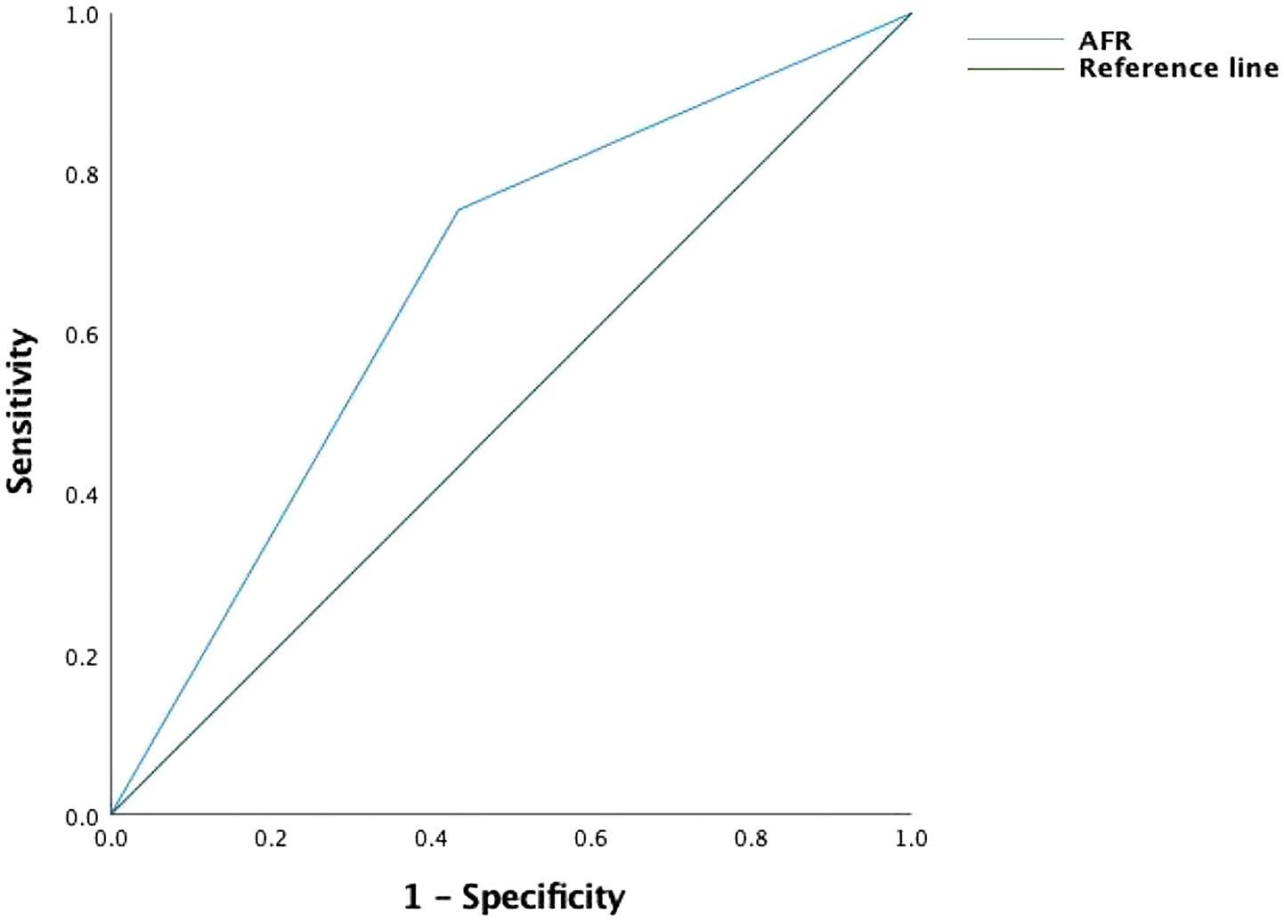
Receiver operating characteristic curve of albumin‐fibrinogen ratio for predicting 3‐year overall survival in patients with hypopharyngeal cancer after treatment.

**FIGURE 2:**
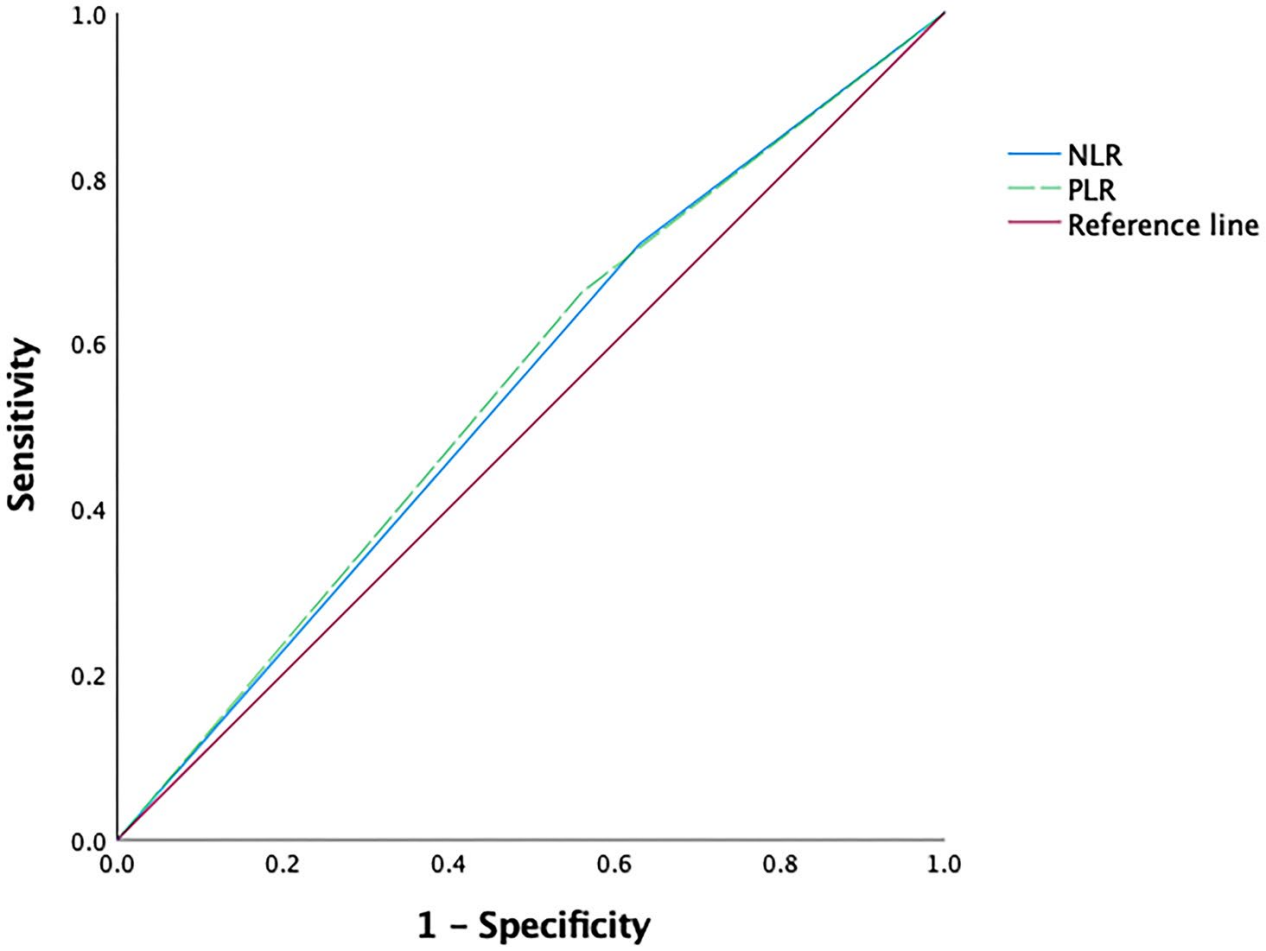
Receiver operating characteristic curve of neutrophil‐lymphocyte ratio (NLR) and platelet‐lymphocyte ratio (PLR) for predicting 3‐year overall survival in patients with hypopharyngeal cancer after treatment.

We enrolled 193 LA‐HPSCC patients in this study, including 179 male patients and 14 female patients. Among them, 37 patients had stage III disease, and 156 patients had stage IV disease (stage IVA, 125 patients; stage IVB, 31 patients). At the time of presentation, 30 (15.5%) patients had a second primary tumor (esophageal cancer, 21 patients; gastric and cardia cancer, 3 patients; lung cancer, 3 patients; and other tumors, 3 patients); during the follow‐up, another 12 patients developed a second primary cancer (esophageal cancer, 3 patients; gastric and cardia cancer, 4 patients; lung cancer, 2 patients; and other tumors, 3 patients). The median RT dose was 66 Gy (range: 50.4–72.6 Gy), and 20 patients received doses < 66 Gy (> 63 Gy in 7 patients; 63 Gy in 4 patients; 60 Gy in 7 patients; and < 60 Gy in 2 patients). The optimal cutoff points for AFR, PLR, and NLR before RT were determined using ROC curves with the maximum value of the Youden index (sensitivity + specificity − 1). The cutoff values of AFR, PLR, and NLR were 10.33, 128.05, and 2.18, respectively, and the areas under the ROC curves were as follows: AFR, 0.673 (95% confidence interval [CI]: 0.590–0.755, Figure 1); PLR, 0.549 (95% CI: 0.459–0.639); and NLR, 0.510 (95% CI: 0.416–0.602; Table 1, Figure 2).

**TABLE 1:** Clinical characteristics of patients with locally advanced hypopharyngeal squamous cell carcinoma (*n* = 193).

Characteristic	Number of patients
Gender	
Male	179
Female	14
Age	
≤ 65 years	121
> 65 years	72
Smoking	
Yes	137
No	56
Drinking	
Yes	116
No	77
Second primary cancer	
Yes	30
No	163
Radiation dose	
≥ 66 Gy	173
< 66 Gy	20
Clinical T stage	
T1	16
T2	60
T3	78
T4	39
Clinical N stage	
N0	21
N1	22
N2	125
N3	25
Clinical TNM stage	
III	37
IVA	125
IVB	31
Lesion site	
Pyriform fossa	133
Posterior cricoid	44
Posterior pharyngeal wall	16
Albumin‐fibrinogen ratio	
> 10.33	102
≤ 10.33	91
Platelet‐lymphocyte ratio	
> 128.05	122
≤ 128.05	71
Neutrophil‐lymphocyte ratio	
> 2.18	134
≤ 2.18	59

### 3.2 Treatments

All patients underwent IMRT, including 23 who received IMRT alone and 170 who received IMRT and chemotherapy. Among them, 80 patients received IC, 43 patients received IC and CCRT, 39 patients received CCRT, and 8 patients received AC after IMRT. Of the 193 patients, 177 completed IMRT, and 16 patients did not complete IMRT. The IC regimens included taxane‐platinum/taxane‐carboplatin (TP/TC, 58 patients), taxane‐platinum‐fluorouracil (TPF, 8 patients), platinum‐fluorouracil (PF, 32 patients), fluorouracil‐leucovorin‐platinum (FLP, 23 patients), and gemcitabine‐platinum (GP, 2 patients), and 1, 2, 3, and > 3 cycles of IC were completed in 34, 75, 8, and 6 patients, respectively. The platinum‐based agents used were cisplatin, carboplatin, oxaliplatin, nedaplatin, and lobaplatin. Among those who received CCRT, 58 patients received TP/TC, 12 received FLP, 6 received PF, 2 received paclitaxel, and 4 received platinum‐based agents. In total, 57, 18, and 2 patients completed 1, 2, and 3 cycles of chemotherapy during IMRT, respectively, and 5 patients had weekly chemotherapy cycles during IMRT. Among all the patients who received chemotherapy, 46 patients received TP/TC, 18 received FLP, 12 received TPF/PF, 2 received paclitaxel as a single agent, 1 received etoposide‐cisplatin (EP), and 1 patient received tegafur as a single agent. The median IMRT dose was 66 Gy (range: 50.4–72.6 Gy). The radiation dose was ≥ 66 Gy in 173 patients and < 66 Gy in 20 patients.

### 3.3 Overall Survival

By the end of the follow‐up (October, 31, 2022), 57 patients were still surviving, and 136 patients had died. The causes of death were as follows: local progression or regional lymph node metastasis (61 patients); distant metastasis (14 patients), including lung metastasis (7 patients), liver metastasis (2 patients), bone metastasis (1 patient), and multiple metastases (4 patients); other tumors (16 patients); non‐tumor‐related causes (10 patients); and other causes such as cachexia and unknown cause of death (35 patients).

**FIGURE 3:**
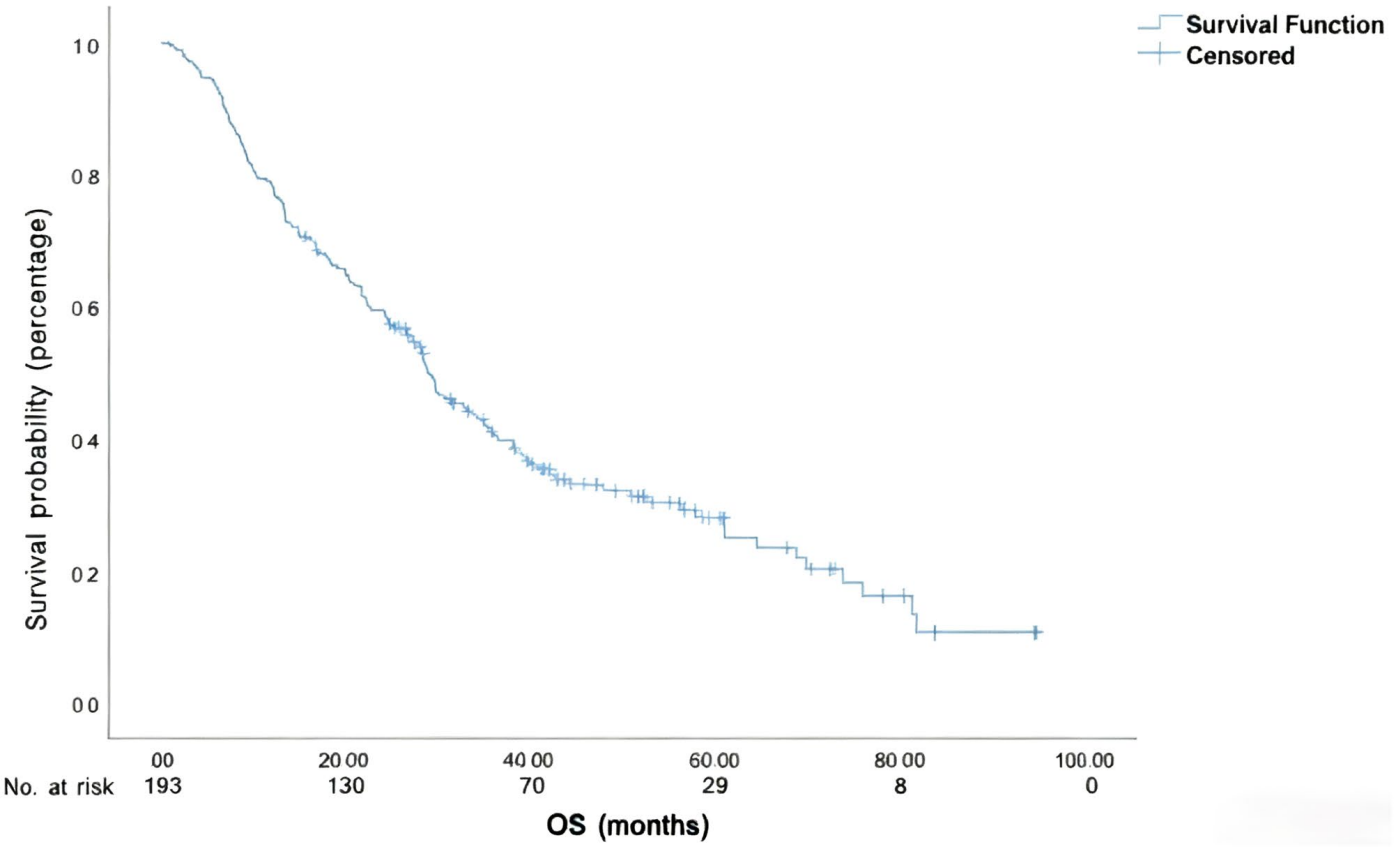
Overall survival (OS) in patients with hypopharyngeal cancer after treatment.

The median OS duration was 28.9 months, and 1‐year, 2‐year, and 3‐year OS rates for the entire cohort were 78.2%, 59.4%, and 41.1%, respectively (Figure 3). Univariate analysis showed that AFR, PLR, NLR, second primary cancer, clinical T (cT) stage, clinical N (cN) stage, treatment modality, and short‐term response to treatment (assessed at 1 month after treatment using RECIST v1.1 criteria) were associated with OS (Table 2). Sex, age, AFR, PLR, NLR, second primary cancer, cT stage, cN stage, short‐term treatment response, and treatment modality were entered into multivariate Cox analysis, and cT stage, second primary cancer, AFR, and short‐term treatment response were found to be independent risk factors for OS (Table 3).

**TABLE 2:** Univariate analysis of factors influencing OS in LA‐HPSCC patients.

Factor	Patients (*n* = 193)	OS			*χ* ^2^	*p*
		Median (months)	1‐year (%)	3‐year (%)		
Gender						
Male	179	28.9	77.7	41.2	0.053	0.818
Female	14	29.9	85.7	38.6		
Age (years)						
> 65	72	28.5	72.2	38.8	1.994	0.158
≤ 65	121	29.9	81.8	42.4		
Smoking						
No	56	28.0	78.6	36.1	2.098	0.147
Yes	137	30.2	78.1	43.1		
Drinking						
No	77	29.6	80.5	42.0	0.007	0.931
Yes	116	28.9	76.7	40.4		
Lesion site						
Pyriform fossa	133	29.2	78.2	41.2	0.492	0.782
Posterior cricoid	16	38.3	81.3	56.3		
Posterior pharyngeal wall	44	28.0	77.3	34.9		
Second primary cancer						
Yes	30	29.8	79.8	43.4	3.890	0.049
No	163	24.4	70.0	28.7		
cT stage						
T1	16	44.2	87.5	73.9	14.800	0.002
T2	60	28.5	80.0	43.5		
T3	78	29.9	79.5	44.3		
T4	39	20.0	69.2	17.9		
cN stage						
N0	21	30.9	85.7	49.0	3.483	0.323
N1	22	57.9	90.9	60.6		
N2	125	28.4	76.8	38.4		
N3	25	16.8	68.0	32.0		
cTNM stage						
III	37	60.9	89.2	61.7	6.352	0.042
IVA	125	28.4	78.4	38.3		
IVB	31	16.8	64.5	29.0		
Short‐term response						
CR + PR	147	33.8	79.6	47.5	10.178	0.001
SD + PD	46	21.1	73.9	18.9		
Treatment modality						
CCRT	39	38.1	87.2	57.2	8.802	0.032
IC + CCRT	44	50.8	77.3	52.0		
IC + IMRT	79	28.8	83.5	32.2		
IMRT alone/IMRT + AC	31	18.0	54,8	32.3		
AFR						
≤ 10.33	91	22.2	70.3	28.8	18.667	0.000
> 10.33	102	39.2	85.3	52.4		
PLR						
≤ 128.05	71	36.1	77.5	50.1	5.010	0.025
> 128.05	122	28.0	77.9	34.4		
NLR						
≤ 2.18	59	33.1	81.4	46.8	3.860	0.049
> 2.18	134	28.3	75.4	37.7		

Abbreviations: AC, adjuvant chemotherapy; AFR, albumin‐fibrinogen ratio; CCRT, concurrent chemoradiotherapy; CR, complete response; IC, induction chemotherapy; IMRT, intensity‐modulated radiotherapy; LA‐HPSCC, locally advanced hypopharyngeal squamous cell carcinoma; NLR, neutrophil‐lymphocyte ratio; OS, overall survival; PD, progressive disease; PLR, platelet‐lymphocyte ratio; PR, partial response; SD, stable disease.

**TABLE 3:** Multivariate analysis of factors influencing OS in LA‐HPSCC patients.

Factor	HR (95% CI)	*p*
Second primary cancer		
No	1	0.025
Yes	1.665 (1.065–2.602)	
Short‐term response		
CR + PR	1	0.001
SD + PD	1.901 (1.288–2.804)	
AFR		
10.33	1	0.000
≤ 10.33	1.952 (1.372–2.778)	
cT stage		
T1	1	0.012
T2	1.681 (0.813–3.477)	
T3	1.509 (0.740–3.077)	
T4	2.841 (1.335–6.046)	

Abbreviations: AFR, albumin‐fibrinogen ratio; CI, confidence interval; CR, complete response; HR, hazard ratio; LA‐HPSCC, locally advanced hypopharyngeal squamous cell carcinoma; OS, overall survival; PD, progressive disease; PR, partial response; SD, stable disease.

### 3.4 Progression‐Free Survival

By the end of the follow‐up, 90 patients had developed tumor progression, including 73 patients with primary tumor recurrence or regional lymph node metastasis, and 17 patients with distant metastasis (8 patients with lung metastasis, 3 patients with liver metastasis, 1 patient with bone metastasis, 1 patient with axillary lymph node metastasis, and 4 patients with multiple metastases).

**FIGURE 4:**
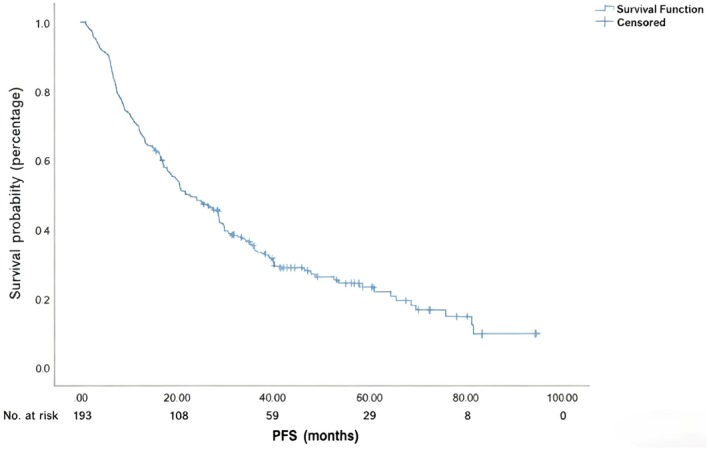
Progression‐free survival (PFS) in patients with hypopharyngeal cancer after treatment.

The median PFS duration was 21.8 months, and the 1‐year, 2‐year, and 3‐year PFS rates for the entire cohort were 69.9%, 49.4%, and 34.7%, respectively (Figure 4). Univariate analysis showed that cT stage, AFR, PLR, treatment modality, and short‐term treatment response were associated with PFS (*p* < 0.05; Table 4), while cN stage, clinical TNM stage, NLR, gender, age, smoking history, alcohol history, and tumor location were not related to PFS (*p* > 0.05). Multivariate analysis included gender, age, cT stage, cN stage, AFR, PLR, NLR, short‐term treatment response, and treatment modality. The results showed that AFR and short‐term treatment response were independent risk factors for PFS (Table 5).

**TABLE 4:** Univariate analysis of factors influencing PFS in patients with LA‐HPSCC.

Factor	Patients	PFS			χ^2^	*p*
		Median (months)	1‐year (%)	3‐year (%)		
Gender						
Male	179	20.6	68.7	34.3	0.298	0.585
Female	14	29.9	85.7	38.6		
Age (years)						
> 65	72	25.2	72.2	34.6	0.341	0.559
≤ 65	121	20.6	68.6	34.7		
Smoking						
No	56	21.8	71.4	30.0	0.876	0.349
Yes	137	22.8	69.3	36.4		
Drinking						
No	77	25.2	74.0	32.8	0.060	0.806
Yes	116	20.3	67.2	35.9		
Lesion site						
Pyriform fossa	133	25.0	70.7	36.4	0.576	0.750
Posterior cricoid	16	18.0	75.0	35.0		
Posterior pharyngeal wall	44	17.3	65.9	28.9		
cT stage						
T1	16	40.2	81.3	61.1	10.201	0.017
T2	60	27.3	73.3	41.9		
T3	78	20.7	73.1	31.9		
T4	39	12.5	53.8	18.3		
cN stage						
N0	21	30.9	81.0	49.0	3.979	0.264
N1	22	26.8	86.4	43.3		
N2	125	20.6	67.2	31.1		
N3	25	13.8	56.0	32.0		
cTNM stage						
III	37	36.5	86.5	51.6	5.115	0.078
IVA	125	20.6	69.6	31.0		
IVB	31	13.2	51.6	29.0		
Short‐term response						
CR + PR	147	28.6	75.5	40.6	14.144	0.000
SD + PD	46	13.4	52.2	15.9		
Treatment modality						
CCRT	39	36.1	76.9	51.1	8.058	0.045
IC + CCRT	44	30.9	70.5	42.9		
IC + IMRT	79	20.4	72.2	26.5		
IMRT alone/IMRT + AC	31	16.0	54.8	25.8		
AFR						
≤ 10.33	91	16.4	57.1	24.5	16.284	0.000
> 10.33	102	32.6	81.4	45.0		
PLR						
≤ 128.05	71	29.8	74.6	39.7	4.063	0.044
> 128.05	122	20.3	66.4	29.8		
NLR						
≤ 2.18	59	28.8	79.7	36.0	3.199	0.074
> 2.18	134	19.5	64.9	33.4		

Abbreviations: AC, adjuvant chemotherapy; AFR, albumin‐fibrinogen ratio; CCRT, concurrent chemoradiotherapy; CR, complete response; IC, induction chemotherapy; IMRT, intensity‐modulated radiotherapy; LA‐HPSCC, locally advanced hypopharyngeal squamous cell carcinoma; NLR, neutrophil‐lymphocyte ratio; PD, progressive disease; PFS, progression‐free survival; PLR, platelet‐lymphocyte ratio; PR, partial response; SD, stable disease.

**TABLE 5:** Multivariate analysis of factors influencing PFS in patients with LA‐HPSCC.

Factor	HR (95% CI)	*p*
Short‐term efficacy		
CR + PR	1	0.001
SD + PD	2.134 (1.465–3.109)	
AFR		
10.33	1	0.000
≤ 10.33	2.020 (1.452–2.809)	

Abbreviations: AFR, albumin‐fibrinogen ratio; CI, confidence interval; CR, complete response; HR, hazard ratio; LA‐HPSCC, locally advanced hypopharyngeal squamous cell carcinoma; PD, progressive disease; PFS, progression‐free survival; PR, partial response; SD, stable disease.

### 3.5 Treatment Modality and Patient Survival

Subgroup analysis showed that the OS did not differ between the CCRT group and the IC + CCRT group (*p* > 0.05), but the OS was significantly higher in the CCRT group and the IC + CCRT group than in the RT alone group (*p* ≤ 0.05). The OS was higher in the IC + CCRT group than in the IC + RT group (*p* < 0.05). The OS was higher in the CCRT group than in the IC + RT group (*p* < 0.05). Next, we divided the patients into a group with CCRT (including the IC + CCRT group and CCRT group) and a group without CCRT (including the IC + RT group and RT + AC group). Compared with patients without CCRT, patients with CCRT had significantly improved OS and PFS (*p* < 0.05). Further analysis showed that the improvements in OS and PFS relative to the patients without CCRT mainly occurred in CCRT‐group patients who were aged ≥ 70 years, had cN2/N3 disease, or had cTNM stage III or IVA disease (*p* < 0.05). In contrast, the OS and PFS did not significantly differ between patients who received CCRT and those who did not receive CCRT when they were aged < 70 years, had any cT or cN0/N1, or had stage IVB disease (*p* > 0.05; Tables 6 and 7).

**TABLE 6:** OS in LA‐HPSCC patients with or without CCRT.

Factor	CCRT	Patients	OS			*χ* ^2^	*p*
			Median (months)	1‐year (%)	3‐year (%)		
Age (years)							
≥ 70	No	36	20.0	61.1	25.0	6.808	0.009
	Yes	10	81.2	88.9	77.8		
< 70	No	74	28.9	83.3	33.1	2.039	0.153
	Yes	73	36.5	79.5	52.5		
cT stage							
T1/T2	No	38	28.5	78.9	39.5	3.816	0.051
	Yes	38	69.7	84.2	62.4		
T3	No	48	24.8	79.2	34.8	3.090	0.079
	Yes	30	39.2	80.0	62.1		
T4	No	24	13.5	62.5	16.7	0.146	0.702
	Yes	15	24.2	80.0	20.0		
cN stage							
N0/N1	No	27	35.9	85.2	47.6	3.474	0.062
	Yes	16	73.6	93.8	68.8		
N2/N3	No	83	22.4	72.3	27.2	7.673	0.013
	Yes	67	36.1	79.1	51.0		
cTNM stage							
III + IVA	No	94	27.3	77.7	33.6	10.155	0.001
	Yes	68	39.2	85.5	60.1		
IVB	No	16	16.7	62.5	25.0	0.026	0.873
	Yes	15	16.8	66.7	33.3		

Abbreviations: CCRT, concurrent chemoradiotherapy; LA‐HPSCC, locally advanced hypopharyngeal squamous cell carcinoma; OS, overall survival.

**TABLE 7:** PFS in LA‐HPSCC patients with or without CCRT.

Factor	CCRT	Patients	PFS			*χ* ^2^	*p*
			Median (months)	1‐year (%)	3‐year (%)		
Age (years)							
≥ 70	No	36	16.4	71.1	22.2	5.747	0.025
	Yes	10	81.2	90.0	65.6		
< 70	No	74	18.9	70.3	28.3	2.948	0.086
	Yes	73	34.4	69.9	45.1		
cT stage							
T1/T2	No	38	20.6	73.7	34.2	3.700	0.054
	Yes	38	46.5	76.3	59.1		
T3/T4	No	72	18.0	63.9	22.1	3.073	0.080
	Yes	45	25.2	71.1	36.4		
cN stage							
N0/N1	No	27	29.9	81.5	44.1	1.164	0.281
	Yes	16	30.9	87.5	50.0		
N2/N3	No	83	17.1	62.7	20.5	8.290	0.004
	Yes	67	34.4	68.7	45.7		
cTNM stage							
III + IVA	No	94	20.3	71.3	26.5	8.885	0.003
	Yes	68	36.0	76.5	49.7		
IVB	No	16	8.9	43.8	25.0	0.547	0.459
	Yes	15	16.6	60.0	33.3		

Abbreviations: CCRT, concurrent chemoradiotherapy; LA‐HPSCC, locally advanced hypopharyngeal squamous cell carcinoma; OS, overall survival.

## 4 Discussion

The prognosis of HPC is poor due to its inconspicuous anatomical location and lack of specific symptoms during the early stage. In most patients, the tumor is found to have progressed to the locally advanced stage at the time of diagnosis, resulting in low 5‐year OS rates of 25%–50% among HPC patients [9, 10]. Yang et al. [11] prospectively analyzed 260 patients with locally advanced HPC treated with IC followed by integrated treatment modalities; the 3‐year and 5‐year OS rates were 46.0% and 32.6%, respectively, and the 3‐year and 5‐year PFS rates were 41.0% and 26.6%, respectively. Zhao et al. [12] analyzed 116 patients with locally advanced HPC treated with CCRT; the 3‐year OS rate was 43.1%, and the median PFS and OS durations were 26.1 and 28.0 months, respectively. In our study, among the 193 LA‐HPSCC patients who received definitive RT or CRT, the median OS and PFS durations were 28.9 and 21.8 months, respectively, and the 3‐year OS and PFS rates were 41.1% and 34.7%, respectively. Thus, our results are consistent with those of the aforementioned studies.

Patients with squamous cell carcinoma of the head and neck (SCCHN) often develop a second primary carcinoma (SPC), with reported incidence rates ranging between 14% and 36% [13]. The risk of SPCs in HPC patients is even higher [14]. Yamamoto et al. reported that synchronous or metachronous SPCs were found in 46.3% of the 136 HPC patients in their study [15]. Luo et al. identified that the most common sites for SPCs in HPC patients were the oral cavity, oropharynx, thyroid, esophagus, stomach, and lungs [16]. The prognosis of HPC patients with SPCs remains controversial [17]. Luo et al. analyzed a retrospective cohort of 593 patients with HPC, and found that 73 patients developed metachronous SPCs. However, the authors found no significant differences in survival between HPC patients with and without SPCs (5‐year OS: 47.3% vs. 43.6%, *p* = 0.657) [16]. In contrast, Rennemo et al. reported that 17% of the SCCHN patients in their study developed SPCs, and the median OS rate of patients with SPCs was < 20% [18]. Zhang et al. further emphasized that the presence of SPCs at initial diagnosis was an independent risk factor for reduced OS in HPC patients (*p <* 0.05) [19]. In our study, 21.8% of LA‐HPSCC patients exhibited synchronous or metachronous SPCs, and these predominantly occurred in the esophagus, stomach, and lungs. Notably, the presence of SPCs was correlated with significantly reduced OS. Given the 21.8% incidence rate of SPCs in our cohort—predominantly esophageal, gastric, or lung malignancies—routine surveillance (e.g., endoscopy for high‐risk patients, low‐dose CT for lung screening) enables early detection and intervention. For synchronous SPCs (e.g., hypopharyngeal‐esophageal primaries), multidisciplinary teams should tailor strategies to integrate concurrent RT or prioritize sequential therapy based on tumor aggressiveness and patient fitness [16]. The management of HPCs complicated by SPCs is highly complex, requiring individualized, multidisciplinary approaches. The presence of SPCs—most commonly in the esophagus or lungs—significantly worsens prognosis and complicates treatment planning. For locally advanced HPC, surgery (often total laryngectomy) or CRT is standard. Meta‐analyses and large cohort studies show similar OS between surgery and CRT, but CRT offers better laryngeal preservation, while surgery may provide better disease‐free survival in some subgroups [20, 21]. The presence of SPCs is an independent adverse prognostic factor, significantly reducing OS. Additionally, the T stage of the SPC (especially esophageal cancer) is a key determinant of clinical outcomes, and early detection and intervention are associated with better survival [15]. Surveillance and risk‐reduction strategies are vital to improve outcomes in this high‐risk population.

The TNM staging system serves as a critical prognostic factor in oncology patients. Accumulating evidence shows that advanced T‐stage, advanced N‐stage, distant metastasis, and clinical TNM stage are associated with OS in HPC patients [22, 23]. For instance, Lu et al. retrospectively analyzed 321 HPC patients, and identified that both T‐stage and N‐stage were independent risk factors for OS [23]. Krstevska et al. reported that the T stage was an independent risk factor for OS in patients with locally advanced HPC after CCRT, and a higher T‐stage correlated with poorer survival [24]. Similarly, Joo et al. [25] reported that lymph node metastasis was an independent risk factor for OS in HPC patients. In our study, the cTNM stage emerged as an independent risk factor for OS in LA‐HPSCC patients; this result is aligned with those of the above researchers.

According to the NCCN and CSCO guidelines, the regimen of IC followed by radical RT or CCRT is recommended for patients with locally advanced SCCHN [5, 6]. Studies have indicated that as compared to RT alone, CCRT significantly improves short‐term efficacy and OS in patients with locally advanced SCCHN [26, 27]. In a phase III randomized trial by Prades et al. [28], 75 patients with T3N0 HPC received either CCRT or IC followed by curative RT; the 2‐year OS rates were 47% vs. 51%, respectively, and the rates of laryngeal preservation at 2 years were 92% vs. 68% (*p* = 0.016), respectively, supporting the superiority of CCRT in organ preservation. Another study by Yoon et al. [29] included 66 patients with locally advanced HPC; compared with IC + RT, IC + CCRT significantly improved the 5‐year OS rate (24% vs. 42%, *p* = 0.031) and 3‐year locoregional control rate (23% vs. 52%, *p* = 0.004). In the current study, patients with LA‐HPSCC who received CCRT (CCRT or IC + CCRT) had improved OS and PFS as compared to patients who did not receive CCRT (IC + RT or RT + AC), especially in the case of patients aged ≥ 70 years, and those with stage N2/N3 or stage III or IVA disease.

In recent years, the value of IC in treating HNSCC patients is being increasingly recognized. Cohen et al. [30] carried out a phase III trial that enrolled 285 patients with locally advanced SCCHN, and the results demonstrated that OS did not significantly differ between the IC + CCRT and CCRT‐alone arms (*p* > 0.05). This result is aligned with those of earlier studies by Haddad et al. [31] and Hitt et al. [32]; in cohorts with unresectable locally advanced SCCHN, IC + CCRT failed to augment survival outcomes as compared with CCRT. In contrast, Ghi et al. [33] reported that compared with CCRT, IC followed by cetuximab and concurrent RT or CCRT could increase 3‐year OS (57.5% vs. 46.5%, *p* = 0.031). Conversely, Luo et al. [34] reported that LA‐HPSCC patients treated with docetaxel/cisplatin/5‐fluorouracil‐based IC followed by CCRT and those treated with definitive CCRT showed similar 3‐year OS rates (53.1% vs. 54.8%, *p* = 0.988). In our study, the 3‐year OS rates in the IC + CCRT group and the CCRT group were 52.0% and 53.8%, respectively (*p* = 0.702). The role of IC in LA‐HPSCC patients scheduled to be treated with CCRT warrants further study.

Achieving a complete response (CR) following radical CRT has been consistently associated with improved survival outcomes across multiple malignancies [35]. For instance, a pooled analysis of breast cancer trials (DB‐01, DB‐02, and DB‐03) involving 834 patients revealed substantial benefits in PFS (CR: not estimable; PR: 21.8 months; SD/PD: 8.4 months) and OS (CR: not estimable; PR: 40.5 months; SD/PD: 23.6 months) among patients attaining CR as compared to those achieving a PR or having SD/PD [36]. Similarly, a merged analysis of 2 phase‐III melanoma trials (COMBI‐d and COMBI‐v) demonstrated profound dose‐dependent survival gains [37]. The SD, PR, and CR groups exhibited distinct 5‐year PFS rates (1%, 16%, and 49%, respectively) and OS rates (16%, 32%, and 71%, respectively), underscoring the prognostic significance of treatment response depth [37]. Our findings align broadly with those of the above studies: the CR/PR rate in this study was 76.2% (147/193), and the median OS was significantly higher in the CR/PR group than in the SD + PD group (33.8 vs. 22.1 months, *p* = 0.001).

A growing body of evidence has highlighted systemic inflammation as a pivotal driver of tumor progression. NLR, PLR, albumin levels, and C‐reactive protein have emerged as prognostic indicators in head‐and‐neck cancers [38, 39, 40, 41]. Ruiz‐Ranz et al. retrospectively analyzed the data of 348 patients with oral squamous cell carcinoma treated with surgery, and identified that elevated preoperative NLR independently predicted poor OS (hazard ratio [HR] = 1.626, 95% confidence interval [CI]: 1.004–2.633, *p* = 0.04) [39]. Consistent with this, Zeng et al. reported that a higher baseline PLR was correlated with poor survival in nasopharyngeal carcinoma patients (HR = 3.480, 95% CI: 1.090–11.117; *p* = 0.035) [38]. In our study, univariate analysis indicated that NLR (*p* = 0.025) and PLR (*p* = 0.049) were related to OS in LA‐HPSCC patients, but these were not independent risk factors for OS. Notably, few reports have explored the role of the albumin‐to‐fibrinogen ratio (AFR) in HNSCC, though the value of AFR as a prognostic indicator has been evaluated in patients with hepatocellular carcinoma [42], colorectal cancer [43], and gastric cancer [44]. Wang et al. reported that AFR was an independent factor for OS and PFS in patients with locally advanced SCCHN [45]. Our study also identified AFR as an independent prognostic marker of both OS and PFS in LA‐HPSCC patients after IMRT (*p* < 0.001). Notably, AFR correlated positively with treatment response, supporting its dual utility in mirroring inflammatory status and nutritional resilience. To our knowledge, this is the first study to validate AFR as an independent prognostic marker for both OS and PFS in LA‐HPSCC patients undergoing radical RT or CRT. This finding extends beyond prior research focused on NLR/PLR, providing a novel biomarker for risk stratification.

The limitations of this study should be acknowledged. First, this is a single‐center, retrospective study, and selection bias cannot be avoided. Second, the administration of adjuvant chemotherapy in 8 patients deviated from standard protocols, primarily due to complex comorbidities (synchronous SPCs in 5 patients, salvage intent after disease progression in 1 patient, and data unavailable in 2 patients). Moreover, the retrospective nature of our study limited our ability to ascertain a precise rationale for these deviations in all patients, reflecting real‐world clinical dilemmas. Third, the heterogeneity in radiation doses (50.4–72.6 Gy), while reflecting real‐world clinical adaptation, may limit direct outcome comparisons. Notably, 2 patients received < 60 Gy: a 79‐year‐old patient with poor performance status (KPS < 60) who was treated palliatively (50.4 Gy), and a patient with a synchronous cervical esophageal cancer who developed grade 3 hemoptysis requiring treatment interruption at 57 Gy. Fourth, some patients failed to complete IMRT; different IC and CCRT regimens were used, and the number of chemotherapy cycles varied among the patients. All these factors may affect treatment response. Fifth, we acknowledge that management strategies for multifocal lesions were not detailed, limiting technical reproducibility. Sixth, this study failed to analyze treatment‐related side effects during IC and CCRT, and detailed reasons for failure and salvage treatments were not analyzed. Only 45 of the 123 patients who received IC had response assessments available after treatment. Among these 45 patients, 16 patients (35.6%) achieved a PR or CR. OS and PFS did not significantly differ between patients who attained CR and those who attained PR after IC. Finally, the sample sizes of some subgroups were small; additionally, the follow‐up period was short, and some patients were not regularly followed up, which may affect the evaluation of disease recurrence or progression. Nonetheless, our analysis of 193 LA‐HPSCC patients establishes this as a major single‐center investigation for this uncommon malignancy, providing comprehensive real‐world evidence from China, and the results of this single‐center real‐world study still have clinical value.

## 5 Conclusion

In conclusion, among patients with LA‐HPSCC treated with RT or CRT, OS and PFS were higher in those with early cTNM stages, high AFR, low PLR, CCRT, and CR + PR after IMRT. Patients with low NLR had higher OS and a higher incidence of second primary cancers (commonly esophageal cancer and lung cancer).

## Author Contributions

**Xinyue Qu:** data curation; formal analysis; writing – original draft. **Zhen Zhao:** conceptualization; writing – review and editing. **Yunfan Wu:** data curation; formal analysis; writing – original draft. **Rong Qiu:** data curation; formal analysis; writing – review and editing. **Meng Song:** data curation; formal analysis; writing – review and editing. **Zhuofan Wang:** data curation; formal analysis; writing – review and editing. **Qi Liu:** data curation; formal analysis; writing – original draft. **Yan Zhao:** conceptualization; writing – review and editing; methodology. **Yuxiang Wang:** conceptualization; writing – review and editing.

## Ethics Statement

The study was approved by the ethics committee of the Fourth Hospital of Hebei Medical University (2022KY262), Shijiazhuang, China. Patient consent was waived since this was a retrospective study.

## Conflicts of Interest

The authors declare no conflicts of interest.

## Data Availability

Data are available upon request to the corresponding author (Dr. Yuxiang Wang).
